# Fatty Acid Profile of Neutral and Polar Lipid Fraction of Wild Eggs and Hatchlings from Wild and Captive Reared Broodstock of *Octopus vulgaris*

**DOI:** 10.3389/fphys.2017.00453

**Published:** 2017-07-24

**Authors:** Juan Estefanell, Antonio Mesa-Rodríguez, Besay Ramírez, Antonio La Barbera, Juan Socorro, Carmen María Hernandez-Cruz, María Soledad Izquierdo

**Affiliations:** ^1^Grupo de Investigación en Acuicultura, Parque Científico Tecnológico Marino, Universidad de Las Palmas de Gran Canaria Las Palmas, Spain; ^2^Ciclo Superior Cultivos Acuicolas, Instituto de Educacion Secundaria les Profesor Cabrera Pérez Las Palmas, Spain

**Keywords:** fatty acids, neutral and polar lipids, *Octopus vulgaris*, hatchlings, eggs, artificial dens, wild and captive reared

## Abstract

The culture of *Octopus vulgaris* is constrained by unsolved problems in paralarvae rearing, mainly associated to the unknown nutritional requirements of this species in early stages. In this article we studied the fatty acid profile (total, neutral, and polar lipid fractions) in wild eggs and wild hatchlings, collected in Gran Canaria (SW) (Spain) with artificial dens, in comparison to hatchlings obtained in captivity from broodstock fed on trash fish species. Total lipids were 11.5–13.5% dw, with the polar fraction representing a 70.6–75.5% of total lipid, with lower values in wild hatchling in comparison with captive ones. Docosahexaenoic acid (DHA) was the main component in neutral and polar fatty acid profile in all samples, underlying its importance in this species. Decreasing levels of saturates and arachidonic acid (ARA) from wild eggs to hatchlings, mainly associated to the polar fraction, suggest their use during embryonic development. In hatchlings, increasing levels of oleic acid in the neutral fraction and eicosapentaenoic acid (EPA) in the polar fraction, suggests their importance in hatchlings quality. Wild hatchlings showed in the polar fraction higher oleic acid and ARA, and lower DHA/ARA and EPA/ARA ratios in comparison with captive hatchlings, suggesting a difference in paralarvae nutritional status. These results suggest the importance of n-3 highly unsaturated fatty acids (HUFA), oleic acid, and ARA, presented in the adequate lipid fraction, in the diet of broodstock and paralarvae of *O. vulgaris*.

## Introduction

The common octopus *Octopus vulgaris* is a promising candidate to diversify marine farming for its wide market demand and high growth rates (Vaz-Pires et al., 2004; García García and Cerezo Valverde, 2006; Estefanell et al., 2012a). However, the low survival of the paralarvae after the planktonic phase still constrains the industrial rearing of this species (Iglesias et al., 2007; Iglesias and Fuentes, 2014). To date, best paralarvae growth and survival were obtained when crab zoeas were added as a complement to *Artemia* (Villanueva, 1994, 1995; Iglesias et al., 2004; Carrasco et al., 2006; Fuentes et al., 2011; Reis et al., 2015; Garrido et al., 2016a; Roo et al., 2017), which suggests that nutrition is the main factor affecting the biological performance of early life stages in this species (Navarro et al., 2014). In order to estimate its nutritional requirements, biochemical analysis of wild hatchlings, wild paralarvae (6–8 days old), wild juveniles, and potential natural preys have been performed. In general, these wild individuals showed high phospholipids and high n-3 HUFA (EPA, DHA) and n-6 HUFA (ARA) content (Navarro and Villanueva, 2000, 2003; Estefanell et al., 2013; Garrido et al., 2016b; Roo et al., 2017), abundance in certain amino acids (lysine, leucine, arginine, glutamate, aspartate) (Villanueva et al., 2004) and high levels in some microelements (copper, calcium, strontium, sulfur) (Villanueva and Bustamante, 2006). However, in comparison with these estimated nutritional requirements of paralarvae, the enriched *Artemia* successfully used as live prey in marine fish larval rearing, shows low polar lipid content and an imbalance in the n-3 and n-6 HUFA fatty acid profile (Navarro and Villanueva, 2000; Estefanell et al., 2013; Reis et al., 2015; Garrido et al., 2016b; Roo et al., 2017). Even though *Artemia* enrichment in DHA and polar lipids was attained using marine lecitine (Guinot et al., 2013a), the rapid bioconversion of DHA from the polar to the neutral fraction (Guinot et al., 2013b) suggests the inadequacy of *Artemia* as live prey for *O. vulgaris* paralarvae. These findings also underline the importance of the fraction in which the fatty acids are supplied for the successful rearing of this species. For instance, reared paralarvae of *O. vulgaris* showed most n-6 and n-3 HUFA in the polar fraction and most monoenes in the neutral fraction after 10–30 days of feeding (Navarro and Villanueva, 2003; Viciano et al., 2011). However, no data is available regarding the fatty acid profile of the neutral and polar lipid fractions in eggs and hatchlings, which represents useful information to estimate the nutritional requirements of early stages, contributing to the improvement of enrichment protocols for *Artemia* and specific compound microdiets.

In recent years, new data has been published on the ecology of *O. vulgaris* paralarvae from the NW Atlantic cost of the Iberian peninsula. For instance, decapod crab zoeas were identified as main natural preys by molecular methods (Roura et al., 2012), an oceanic life strategy far from the shelf in paralarvae was observed (Roura et al., 2016) and a preference for spawning areas with hard bottom substrate and moderate depth (<20 m) was detected (Guerra et al., 2015). However, data regarding the initial biochemical profile of wild paralarvae and eggs of *O. vulgaris* is still scarce, which could provide useful information regarding the nutritional requirement in early stages. In particular, one wild egg mass was analyzed from the Mediterranean sea (Navarro and Villanueva, 2003). To our knowledge, only the fatty acid profile from total lipids were obtained in 10 wild paralarvae of 6–8 days old in NW Spain (Garrido et al., 2016b) and in two samples of wild hatchlings and egg masses at the Canary Islands (Estefanell et al., 2013). Generally, the egg of marine species contains all the nutrients that the larvae require during the lecithotrophic phase, prior to exogenous feeding, and is related to the broodstock diet (Mourente and Vazquez, 1996). In *O. vulgaris*, an effect of the broodstock diet was observed on the biochemical profile of gonads (ovary and testis) (Estefanell et al., 2015), eggs and hatchlings (Quintana et al., 2015). Also, in a recent rearing trial with paralarvae of *O. vulgaris*, stress and nutritional condition biomarkers showed significant variability associated to geographical origin, despite applying the same feeding protocol and diet (Garrido et al., 2017). These authors concluded that further research must be carried out in order to understand the physiology of *O. vulgaris* associated to different geographical origins. Indeed, differences in the fatty acid profile were observed in the ovary of wild *O. vulgaris* collected from the natural environment in distant areas (Rosa et al., 2004; Sieiro et al., 2006; Lourenço et al., 2014; Estefanell et al., 2015), probably related to differences in the natural diet (Hanlon and Messenger, 1996). For these reasons, samples of wild eggs and wild hatchlings from different areas must be collected and analyzed in order to obtain information on the nutritional requirements of this species, and search for potential regional differences.

In this study we used indirect methods to obtain information about the neutral and polar fatty acid nutritional requirements in early stages in *O. vulgaris*. For this, we collected wild egg masses in Gran Canaria (Canary Islands, Spain) from the natural environment and obtained wild hatchlings at the lab. Also, we obtained hatchlings from captive broodstock fed on trash fish species commonly used during the grow out phase (Estefanell et al., 2012b).

## Materials and methods

### Ethics in animal research

The protocols for handling and rearing of broodstock of *O. vulgaris*, as well as the protocol for paralarvae euthanasia were approved by the Committee of Ethics in Animal Welfare of the University of Las Palmas de Gran Canaria in compliance with Directive 2010/63/EU.

### Wild eggs and hatchlings

To obtain wild eggs and wild hatchlings, artificial dens were specifically designed to capture females caring eggs. For this, a black “T” shaped PVC 160 mm diameter pipe, with two ends closed with a PVC lid, was attached to a concrete base of 60 × 40 × 15 cm, weighing ~15 kg. Several dens were placed at 10–20 m depth in rocky areas (with abundant crevices and holes) in the SW coasts of Gran Canaria (Las Palmas, Canary Islands). In November, several artificial dens were spotted with eggs. In total, three artificial dens with the female and the egg mass were carefully placed in a 250 L tank to be transported, by boat to the nearest harbor and by car to the ULPGC aquaculture facilities. In total, transport took ~1 h.

Upon arrival to the facility, each den with the female and the eggs was placed individually in 500 L circular tanks, using 5 μm filtered natural seawater (37 ppt) in an open flow through system adjusted to a renovation of 50%/h. Natural photoperiod (November–December) were used during embryonic development. Each tank was covered with a shadowing net and the females were not fed during this period (~1 month). Once the paralarvae started hatching the renovation was reduced to 50 L/h, and the newly hatched paralarvae were retained by a filter (net mesh of 375 μm) in a nearby 100 L tank connected to the 500 L tank. Hatchlings were daily collected (8:00 a.m.). The water temperature was ranged 20–22°C and the oxygen levels were above the 90% saturation.

### Captivity hatchlings

Wild specimens of *O. vulgaris* were provided by professional fishermen and transported to ULPGC aquaculture facilities (Telde, Las Palmas, Canary Islands) in the conditions described by Estefanell et al. (2012b). Subadults of *O. vulgaris*, males:female sex ratio 1:1 (*N* = 6, initial weight: 975 ± 128 g) were kept under social conditions in 1.5 m^3^ rectangular tanks under natural photoperiod (September–October), using 5 μm filtered natural seawater (37 ppt) in an open flow through system adjusted to a renovation of 100%/h. The tank was provided with 12 dens (PVC tubes of 160 mm diameter and 50 cm length) and covered with a shadowing net. During the rearing period the specimens were fed *ad libitum* once a day (six times/week) with fresh bogue *Boops boops* (Estefanell et al., 2012b). The males were removed after 2 weeks. The remaining females naturally spawned after ~2 months. Same methodology as described above was used to collect hatchlings.

### Paralarvae euthanasia protocol

Hatchlings were anesthetized by immersion in seawater with a 1.0% ethanol (96%) for 5 min, prior to being sacrificed by immersion on iced seawater. The same protocol was applied for eggs.

### Dry weight determinations

The hatching period lasted 2–3 weeks. For each female, dry weight (dw) of hatchlings was determined four times during the hatching period. For each time, 30 paralarvae were randomly selected and separated in 3 pools of 10 paralarvae. After being sacrificed, the hatchlings were rinsed with distilled water, prior to being carefully placed on a crystal slide. The dry weight was determined by drying them at 105°C until constant weight.

### Biochemical samples

The following samples were taken: a sample of eggs (3 strings) from each artificial den was taken upon arrival to the aquaculture facility (“wild eggs,” *N* = 3, from different females), hatchlings from the natural environment (“wild hatchlings,” *N* = 3, hatched from eggs from the same females) and hatchlings from broodstock fed on trash fish species under common aquaculture conditions (“captive hatchlings,” *N* = 3, from different females). For each female, ~1,000 hatchlings were sacrificed four times during the hatchling period, and mixed to obtain an homogeneous pool sample (~4 g wet weight). After being sacrificed, the eggs and the hatchlings were rinsed with distilled water to remove ethanol traces, dried on absorbent paper and immediately frozen at −80°C.

### Biochemical analysis

Proximate composition of eggs and hatchlings were analyzed following standard procedures (AOAC, 1997). Moisture was determined after drying the sample in an oven at 105°C to constant weight; ash by combustion in a muffle furnace at 600°C for 12 h; protein content (N × 6.25) was determined by Kjeldahl method and crude lipid was extracted following the method described by Folch et al. (1957). Neutral and polar fractions of total lipids were separated by adsorption chromatography on silica cartridges (Sep-pak; Waters S.A., Massachussets, USA) using 30 mL chloroform and 20 mL chloroform/methanol (49: 1, v/v) as solvent for neutral lipid, followed by a 30 mL methanol wash to obtain the polar fractions according to Juaneda and Rocquelin (1985). Fatty acids methyl esters from total, neutral, and polar lipids were extracted by transmethylation as described by Christie (1982) and separated by gas chromatography under the conditions described by Izquierdo et al. (1992). All analyses were conducted in triplicates.

### Statistical analysis

All data, presented as mean ± standard deviation, were tested for normality (Kolmogorov Smirnov) and homogeneity of variances (Levene's test). When necessary, an arcsin transformation of the data was carried out, particularly when data was presented as % (Fowler et al., 1998). The dry weight of wild and captive hatchlings was compared using a Student “t” model. The proximate composition, neutral, and polar lipid proportions, as well as the % of fatty acid from total, neutral, and polar lipids of wild eggs, wild hatchlings and captive hatchlings were submitted to a one way ANOVA test. In addition, differences among groups were determined with a Tukey *post-hoc* test. When normality or homogeneity of variances was not achieved, non-parametric tests were used (Kruskal Wallis, Games-Howell). In all this manuscript significant differences were considered when *P* < 0.05. For the different analysis, the statistical computer package SPSS v15 (SPSS, Chicago, IL, USA) was used.

## Results

### Proximate composition and fatty acid profile from total lipids

A lower lipid content (% dw) and a higher ash content (%) were observed in eggs (wild) in comparison with hatchlings (*P* < 0.05) (Table 1). Similar lipid and protein content was observed in wild and captive hatchlings, while ash content was higher in wild than in captive hatchlings (*P* < 0.05) (Table 1).

**Table 1:** Dry weight of hatchlings (mg), proximate composition (lipids, proteins, moisture, ash) (%), and main fatty acids profile (% of total fatty acids) from total lipids in wild eggs (*N* = 3), wild hatchlings (*N* = 3), and hatchlings obtained in the lab from captive broodstock fed on trash fish species (*N* = 3).

	**Eggs (wild)**	**Hatchlings (wild)**	**Hatchlings (captivity)**
Dry weight (mg)	–	0.20 ± 0.02	0.22 ± 0.01
Lipids (%dw)	11.5 ± 0.8^a^	13.3 ± 0.2^b^	13.5 ± 0.5^b^
Proteins (%dw)	72.7 ± 3.2	71.6 ± 2.3	72.0 ± 4.2
Moisture (%)	70.4 ± 2.8^a^	85.4 ± 0.2^b^	86.3 ± 5.2^b^
Ash (%)	1.6 ± 0.1^c^	1.3 ± 0.1^b^	1.0 ± 0.1^a^
14:0 (%)	2.8 ± 0.7^b^	1.1 ± 0.0^a^	0.9 ± 0.1^a^
16:0 (%)	21.6 ± 0.2^b^	17.0 ± 0.1^a^	17.3 ± 1.3^a^
16:1n-7 (%)	0.6 ± 0.1^c^	0.3 ± 0.0^b^	0.2 ± 0.0^a^
18:0 (%)	3.8 ± 0.4	4.3 ± 0.1	3.8 ± 0.1
18:1n-9 (%)	5.8 ± 0.2^a^	9.5 ± 0.1^b^	10.4 ± 0.3^c^
18:1n-7 (%)	3.6 ± 0.4^b^	2.6 ± 0.0^a^	2.4 ± 0.3^a^
18:1n-5 (%)	1.9 ± 0.1^c^	1.5 ± 0.1^b^	0.7 ± 0.1^a^
18:2n-6 (%)	0.2 ± 0.1^b^	0.1 ± 0.0^a^	0.1 ± 0.0^a^
20:1n-9 (%)	3.6 ± 0.4	3.7 ± 0.1	3.7 ± 0.1
20:1n-7 (%)	0.3 ± 0.1^a^	0.6 ± 0.0^b^	0.5 ± 0.0^b^
20:2n-6 (%)	1.3 ± 1.1	0.8 ± 0.0	0.9 ± 0.2
20:4n-6 (%)	13.0 ± 1.2^c^	8.5 ± 0.3^b^	4.5 ± 0.5^a^
20:3n-3 (%)	0.1 ± 0.0^a^	2.0 ± 0.1^b^	2.1 ± 0.1^b^
20:4n-3 (%)	0.1 ± 0.0^a^	0.7 ± 0.2^b^	0.5 ± 0.1^b^
20:5n-3 (%)	9.6 ± 0.8^a^	14.0 ± 0.5^b^	15.6 ± 0.4^c^
22:1n-9 (%)	0.2 ± 0.0^a^	0.6 ± 0.0^b^	0.5 ± 0.0^b^
22:4n-6 (%)	1.4 ± 0.2^b^	1.1 ± 0.1^b^	0.3 ± 0.0^a^
22:5n-6 (%)	1.3 ± 0.2^b^	1.1 ± 0.1^b^	0.6 ± 0.1^a^
22:5n-3 (%)	1.5 ± 0.3^b^	2.2 ± 0.1^c^	1.0 ± 0.0^a^
22:6n-3 (%)	24.7 ± 1.0^a^	25.0 ± 0.1^a^	31.9 ± 1.4^b^
∑ Saturates (%)	28.7 ± 0.8^b^	22.8 ± 0.2^a^	22.2 ± 1.5^a^
∑ Monoenes (%)	16.4 ± 0.8^a^	19.3 ± 0.2^b^	18.6 ± 0.2^b^
∑ n-3 (%)	36.6 ± 1.3^a^	44.4 ± 0.9^b^	51.5 ± 1.6^c^
∑ n-6 (%)	17.3 ± 1.9^c^	11.5 ± 0.4^b^	6.4 ± 0.4^a^
∑ n-9 (%)	6.4 ± 0.1^a^	11.5 ± 0.1^b^	11.9 ± 0.2^c^
∑ n-3 HUFA (%)	36.1 ± 1.4^a^	43.8 ± 0.8^b^	51.1 ± 1.6^c^
DHA/EPA	2.6 ± 0.2^c^	1.8 ± 0.1^a^	2.1 ± 0.1^b^
DHA/ARA	1.9 ± 0.2^a^	3.0 ± 0.1^b^	7.1 ± 0.3^c^
EPA/ARA	0.8 ± 0.1^a^	1.6 ± 0.1^b^	3.4 ± 0.1^c^

*All variables are shown as mean ± SD. No significant difference were found in dry weight in hatchlings regardless of origin (P < 0.05). Different superscript letter within a row denotes significant difference among samples (P < 0.05). The ∑ included all detected fatty acids. Selected FA represented 95–98% of total FA*.

Regarding the fatty acids from total lipids, a decrease in saturates (tetradecanoic acid, 14:0; palmitic acid, 16:0) and n-6 (ARA, 20:4n-6), and an increase in monoenes (oleic acid, 18:1n-9) and n-3 HUFA (ETE, 20:3n3; EPA, 20:5n-3) were observed in hatchlings regardless of origin, in comparison with eggs (wild) (*P* < 0.05) (Table 1). Also, a higher ARA and lower DHA content were observed in wild hatchlings in comparison with those obtained from captive broodstock fed on trash fish species (*P* < 0.05) (Table 1).

### Neutral and polar lipid proportion from total lipids and fatty acid profile from each fraction

In general, a higher polar lipid proportion was observed in comparison with the neutral fraction regardless of sample (*P* < 0.05). In particular, the neutral and polar fraction represented a 25.6 ± 0.5 and a 74.4 ± 0.5% in eggs (wild), a 29.4 ± 1.6 and a 70.6 ± 1.6% in hatchlings (wild), and a 24.8 ± 2.5 and a 75.2 ± 2.5% in hatchlings (captivity), respectively (Tables 2, 3). A higher neutral lipid proportion and a lower polar lipid proportion were observed in wild hatchlings in comparison with hatchlings obtained from captive broodstock (*P* < 0.05) (Tables 2, 3).

**Table 2:** Neutral lipids from total lipids (%) and main fatty acids in the neutral fraction in wild eggs (*N* = 3), wild hatchlings (*N* = 3), and hatchlings obtained in the lab from captive broodstock fed on trash fish species (*N* = 3).

	**Eggs (wild)**	**Hatchlings (wild)**	**Hatchlings (captivity)**
Neutral Lipids/Total lipids (%)	25.6 ± 0.5^ab^	29.4 ± 1.6^b^	24.8 ± 2.5^a^
14:0 (%)	5.5 ± 1.3^b^	2.0 ± 0.4^a^	2.1 ± 0.7^a^
16:0 (%)	16.7 ± 1.0	17.3 ± 1.8	15.3 ± 2.1
16:1n-7 (%)	0.9 ± 0.2	0.8 ± 0.3	0.9 ± 0.2
18:0 (%)	4.1 ± 0.3^a^	7.9 ± 0.9^b^	8.8 ± 0.2^b^
18:1n-9 (%)	9.4 ± 0.8^a^	20.7 ± 2.5^b^	16.8 ± 0.4^b^
18:1n-7 (%)	2.5 ± 0.3^b^	2.1 ± 0.2^b^	1.2 ± 0.0^a^
18:1n-5 (%)	0.5 ± 0.0	1.4 ± 1.2	0.2 ± 0.0
18:2n-6 (%)	0.8 ± 0.1^b^	0.1 ± 0.1^a^	0.2 ± 0.3^ab^
20:1n-9 (%)	7.2 ± 0.5^b^	2.5 ± 0.6^a^	3.1 ± 0.4^a^
20:1n-7 (%)	1.5 ± 0.1^b^	0.4 ± 0.0^a^	0.4 ± 0.0^a^
20:2n-6 (%)	0.9 ± 0.1	0.4 ± 0.5	1.4 ± 0.4
20:4n-6 (%)	7.9 ± 1.0^b^	4.4 ± 1.1^a^	4.1 ± 0.1^a^
20: 3n-3 (%)	0.6 ± 0.1^a^	2.0 ± 0.8^b^	1.4 ± 0.3^b^
20:4n-3 (%)	0.2 ± 0.0^a^	0.3 ± 0.2^a^	4.0 ± 0.8^b^
20:5n-3 (%)	8.7 ± 0.5	8.5 ± 0.9	7.2 ± 1.3
22:1n-9 (%)	1.0 ± 0.1	1.0 ± 0.7	1.5 ± 0.4
22:4n-6 (%)	0.8 ± 0.8	1.1 ± 0.7	0.4 ± 0.2
22:5n-6 (%)	1.1 ± 0.3^b^	0.9 ± 0.6^b^	0.1 ± 0.2^a^
22:5n-3 (%)	2.2 ± 0.5^b^	1.5 ± 0.8^ab^	0.6 ± 0.2^a^
22:6n-3 (%)	21.6 ± 2.5^b^	16.9 ± 2.3^a^	21.7 ± 1.4^ab^
∑ Saturates (%)	27.4 ± 2.2	28.7 ± 1.4	27.3 ± 2.1
∑ Monoenes (%)	24.9 ± 2.1^a^	30.6 ± 1.7^b^	25.8 ± 0.9^a^
∑ n-3 (%)	34.1 ± 2.8^ab^	30.6 ± 2.8^a^	36.5 ± 1.7^b^
∑ n-6 (%)	11.8 ± 1.8^b^	7.7 ± 0.2^a^	7.2 ± 0.9^a^
∑ n-9 (%)	12.3 ± 0.9^a^	22.9 ± 2.4^c^	18.8 ± 0.6^b^
∑ n-3 HUFA (%)	33.4 ± 2.9^ab^	29.1 ± 2.3^a^	35.1 ± 1.2^b^
DHA/EPA	2.5 ± 0.2^ab^	2.0 ± 0.5^a^	3.1 ± 0.6^b^
DHA/ARA	2.8 ± 0.5^a^	4.0 ± 1.2^ab^	5.3 ± 0.3^b^
EPA/ARA	1.1 ± 0.2^a^	2.0 ± 0.3^b^	1.8 ± 0.3^b^

*All variables are shown as mean ± SD. Different superscript letter within a row denotes significant difference among samples (P < 0.05). The ∑ included all detected fatty acids. Selected FA represented 92–95% of total FA*.

**Table 3:** Polar lipids from total lipids (%) and main fatty acids in the polar fraction in wild eggs (*N* = 3), wild hatchlings (*N* = 3), and hatchlings obtained in the lab from captive broodstock fed on trash fish species (*N* = 3).

	**Eggs (wild)**	**Hatchlings (wild)**	**Hatchlings (captivity)**
Polar Lipids/Total lipids (%)	74.4 ± 0.5^ab^	70.6 ± 1.6^b^	75.2 ± 2.5^a^
14:0 (%)	2.8 ± 0.9^b^	1.1 ± 0.1^a^	0.9 ± 0.0^a^
16:0 (%)	25.2 ± 1.5^b^	18.1 ± 1.0^a^	20.2 ± 0.3^a^
16:1 n-7 (%)	0.6 ± 0.1^b^	0.1 ± 0.0^a^	0.1 ± 0.1^a^
18:0 (%)	6.6 ± 0.4^b^	4.1 ± 0.4^a^	11.5 ± 0.2^c^
18:1n-9 (%)	3.5 ± 0.6^a^	9.7 ± 0.6^b^	2.6 ± 0.0^a^
18:1n-7 (%)	1.8 ± 0.1^b^	2.5 ± 0.2^c^	0.8 ± 0.0^a^
18:1n-5 (%)	0.4 ± 0.0^b^	1.4 ± 0.0^c^	0.3 ± 0.0^a^
18:2n-6 (%)	0.2 ± 0.0^a^	0.6 ± 0.0^b^	0.6 ± 0.0^b^
20:1n-9 (%)	3.0 ± 0.3^a^	3.4 ± 0.2^ab^	3.8 ± 0.2^b^
20:1n-7 (%)	0.2 ± 0.1^a^	0.6 ± 0.0^b^	0.6 ± 0.1^b^
20:2n-6 (%)	0.7 ± 0.3	0.7 ± 0.1	0.9 ± 0.1
20:4n-6 (%)	13.3 ± 1.0^c^	7.7 ± 0.5^b^	4.5 ± 0.2^a^
20: 3n-3 (%)	0.1 ± 0.0^a^	1.8 ± 0.2^b^	2.1 ± 0.1^b^
20:4n-3 (%)	0.3 ± 0.4	0.2 ± 0.1	0.2 ± 0.0
20:5n-3 (%)	9.3 ± 0.8^a^	12.5 ± 1.3^b^	15.7 ± 1.0^c^
22:1n-9 (%)	0.3 ± 0.1^a^	0.5 ± 0.0^b^	0.6 ± 0.1^b^
22:4n-6 (%)	0.5 ± 0.6	1.4 ± 0.4	0.1 ± 0.2
22:5n-6 (%)	1.2 ± 0.2^ab^	1.6 ± 0.6^b^	0.6 ± 0.1^a^
22:5n-3 (%)	1.2 ± 0.2^a^	3.0 ± 0.8^b^	1.0 ± 0.1^a^
22:6n-3 (%)	22.2 ± 1.2^a^	26.3 ± 2.4^ab^	27.9 ± 1.7^b^
∑ Saturates (%)	35.0 ± 2.2^b^	23.6 ± 1.5^a^	32.9 ± 0.0^b^
∑ Monoenes (%)	10.3 ± 0.8^a^	18.4 ± 1.0^b^	9.0 ± 0.5^a^
∑ n-3 (%)	33.6 ± 1.8^a^	44.1 ± 2.2^b^	47.2 ± 0.3^b^
∑ n-6 (%)	16.3 ± 1.4^c^	12.3 ± 0.4^b^	6.9 ± 0.2^a^
∑ n-9 (%)	4.0 ± 0.5^a^	10.9 ± 0.7^b^	3.9 ± 0.2^a^
∑ n-3 HUFA (%)	33.2 ± 1.8^a^	43.8 ± 2.2^b^	46.9 ± 0.4^b^
DHA/EPA	2.4 ± 0.2	2.1 ± 0.4	1.8 ± 0.2
DHA/ARA	1.7 ± 0.2^a^	3.4 ± 0.5^b^	6.2 ± 0.7^c^
EPA/ARA	0.7 ± 0.1^a^	1.6 ± 0.1^b^	3.5 ± 0.0^c^

*All variables are shown as mean ± SD. Different superscript letter within a row denotes significant difference among samples (P < 0.05). The ∑ included all detected fatty acids. Selected FA represented 92–98% of total FA*.

Regarding the fatty acids from the neutral fraction, the highest monoenes and n-9 were observed in wild hatchlings in comparison with the other samples (*P* < 0.05) (Table 2). In contrast, 20:1n-9 (Eicosenoic acid) and 20:1n-7 (Paullinic acid) showed higher values in eggs in comparison with hatchlings (*P* < 0.05). A decrease in ARA was observed in hatchlings regardless of origin, while EPA showed similar levels in the different samples in the neutral fraction (*P* < 0.05) (Table 2).

Regarding the fatty acids from the polar fraction, the lowest levels of saturates (particularly 18:0, estearic acid) and the highest levels of monoenes and n-9 (mainly associated to oleic acid) were observed in wild hatchlings in comparison with the other samples (*P* < 0.05) (Table 3). A reduction in ARA was observed from wild eggs to wild hatchlings, with hatchling obtained from captive broodstock showing the lowest levels (*P* < 0.05) (Table 3). Increments in EPA, DHA/ARA, and EPA/ARA ratios were observed from wild eggs to wild hatchlings, with hatchling obtained from captive broodstock showing the highest levels (*P* < 0.05) (Table 3). Higher levels of n-3 HUFA (associated to 20:3n3, ETE and EPA) were detected in hatchlings, regardless of origin, in comparison with wild eggs (*P* < 0.05) (Table 3). Wild hatchlings showed higher levels of monoenes (series 18:1n) in comparison with hatchlings from captive broostock (*P* < 0.05) (Table 3).

## Discussion

In this study, *O. vulgaris* hatchlings showed lower dry weights in comparison to data from different regions (0.30–0.48 mg dw; Navarro and Villanueva, 2000; Carrasco et al., 2006; Seixas et al., 2010; Fuentes et al., 2011; Domingues et al., 2013; Iglesias et al., 2014), but similar to previous studies in the Canary Islands (0.17–0.25 mg dw; Reis et al., 2015; Garrido et al., 2017; Roo et al., 2017). This could be related to the higher seawater temperature in Canarian latitudes. Indeed, the incubating temperature is inversely related to hatching size in *O. vulgaris* (Repolho et al., 2014) and in other cephalopod species (*Sepia officinalis, Loligo opalescens, Loligo vulgaris*) (Bouchaud, 1991; Villanueva, 2000; Domingues et al., 2002; Vidal et al., 2002).

Lipid content observed in hatchlings (13.3–13.5% dw) was similar to previous data (Navarro and Villanueva, 2000; Seixas et al., 2010; Iglesias et al., 2014; Roo et al., 2017) and higher than those reported in juveniles and adults of *O. vulgaris* (Navarro and Villanueva, 2003; García García and Cerezo Valverde, 2006; Estefanell et al., 2012b), underlying the importance of the lipid fraction in early stages. This is probably associated to the higher relative size of the digestive gland and especially the nervous and the visual system in hatchlings in comparison with adults. Indeed, lipids in cephalopods are abundant in the digestive gland (García Garrido et al., 2010; Lourenço et al., 2014; Estefanell et al., 2015) and are probably main components of the nervous and visual system, as in several marine fish larvae (Navarro et al., 1995; Benítez-Santana et al., 2007). Regarding the ash content, higher levels were detected in wild eggs and wild hatchlings in comparison with those obtained from captive broodstock, which may have important physiological implications (Davis and Gatlin, 1996). Minerals have several essential functions in cephalopods, such as regulation of acid-base equilibrium and as component of hormones, enzymes and structural proteins, and are affected by fasting conditions (Villanueva and Bustamante, 2006). The analysis of mineral content in wild and reared paralarvae may provide useful information to improve paralarvae survival.

In this study, the proportion of the polar lipid fraction (70–75%) was slightly higher than in previous reports in hatchlings (60–65%) (Navarro and Villanueva, 2000; Quintana et al., 2015; Reis et al., 2015), underlying the importance of the polar fraction in early stages in *O. vulgaris* (Navarro et al., 2014). The importance of the dietary polar lipid fraction has been shown in subadults of this species by its very high digestibility regardless of total dietary lipid content, while the digestibility of the neutral fraction was generally low and inversely related with total dietary lipids (Morillo Velarde et al., 2015). Whether the paralarvae show this selective lipid digestion is unknown. However, low lipid content in crab zoeas (5–10%dw) with high relative levels of phospholipids (Andrés et al., 2010) were suggested to be responsible for the positive effect of these live prey on paralarvae rearing (Iglesias et al., 2014; Reis et al., 2015). Also, the addition of crab zoeas in low quantities to an *Artemia* diet induced a better histological nutritional status of the digestive gland in comparison with paralarvae fed on single *Artemia* (Roo et al., 2017). In contrast, enriched *Artemia* is abundant in lipids (18–28%dw) (Viciano et al., 2011; Iglesias et al., 2014; Roo et al., 2017) and shows a rapid turnover of polar to neutral lipid fraction (Guinot et al., 2013b), inducing negative effects on growth and survival on paralarvae rearing when supplied as a single live prey (Reis et al., 2015; Roo et al., 2017). For these reasons, the supply of the lipids and fatty acids in the adequate fraction appears to be essential for paralarvae rearing success in *O. vulgaris*.

In general, all samples in this study showed high levels of palmitic acid, estearic acid, oleic acid, ARA, EPA, and DHA, in agreement with previous findings (Navarro and Villanueva, 2000, 2003; Quintana et al., 2015; Reis et al., 2015; Roo et al., 2017). The essentiality of ARA, EPA, and DHA in *O. vulgaris* has been suggested by the very low activity of their biosynthesis pathways from n-3 to n-6 substrates (Monroig et al., 2013; Reis et al., 2014). In this study, deviations in the fatty acid profile from total lipids between eggs and hatchlings suggests the use of saturates and ARA during embryonic development, whereas other monoenes and n-3 HUFA are retained or show increasing values in hatchlings. Similar findings were observed in eggs and hatchlings of *O. vulgaris* (Navarro and Villanueva, 2000, 2003). The increase in n-3 HUFA (ETE and EPA) in the polar fraction from wild eggs to wild hatchlings suggest their importance as phospholipids components in paralarvae. The decrease observed in monoenes of the 20:1n series, ARA and DHA in the neutral fraction from wild eggs to wild hatchlings suggest their use as energy substrates during embryonic development. In contrast, increasing values of oleic acid in wild hatchlings, both in the polar and neutral fraction, suggests its importance as energy substrate during the transition from endogenous to exogenous feeding and also as component of phospholipids in paralarvae tissues. Also, the decrease in ARA in the polar fraction in hatchlings is probably associated to a change in phospholipid class, as observed from eggs to hatchlings of *O. vulgaris* fed on different diets (Quintana et al., 2015). In our study, hatchlings obtained from captive broodstock showed a significantly different proportion of polar and neutral lipids and deviations in the fatty acid profile in comparison with wild ones, probably related to the broodstock diet (Quintana et al., 2015). The bogue *Boops boops* used as food shows high DHA and linoleic acid and low ARA content (Estefanell et al., 2012b), mainly provided as triglycerides (neutral lipids) (Cerezo Valverde et al., 2012). In captive hatchlings, the neutral fraction fatty acid profile was relatively similar to wild hatchlings. In contrast, important deviations were observed in the polar fraction, with captive hatchlings showing increasing levels of EPA and the lowest levels of monoenes (18:1n series) and ARA in comparison with wild ones. These variations probably affected the fatty acid profile of the phospholipid classes in hatchlings obtained from captive broodstock (Bell et al., 1995), with potential negative effects on the paralarvae nutritional status. Indeed, different fresh broodstock diets induced differences in spawn quality, related to a change in the fatty acid and the phospholipid class profile in hatchlings (Quintana et al., 2015). In a previous study, important deviations were also observed in the fatty acid profile in gonads (ovary and testis) between wild and reared *O. vulgaris*, especially in the EPA/ARA ratios from total lipids, associated to dietary input (Estefanell et al., 2015). Indeed, difference in the natural diet is probably responsible for the different relation observed in this study among DHA, EPA, and ARA in wild eggs in comparison to previous reports (Navarro and Villanueva, 2003; Estefanell et al., 2013), since cephalopods normally feed on the most readily available prey (Hanlon and Messenger, 1996). Seasonal changes in natural preys and specific oceanographic conditions may explain the important effect of the geographical region on paralarvae rearing success, recently noted in *O. vulgaris* (Garrido et al., 2017).

In the present study, different ratios among ARA, EPA, and DHA in total and polar lipids were observed between wild and captive hatchlings, with several well-known physiological implications in marine species. DHA is especially important in the neural tissue, retina, and the optic nerve which develop during early larval stages in marine fish (Benítez-Santana et al., 2007). In marine fish, ARA and EPA compete with each other for the enzymes that regulate the synthesis of eicosanoids, hormone-like compounds involved in blood clotting, immune and inflammatory response, renal and neural function, cardiovascular tone and reproduction (Tocher, 2003). Also, the deficiency or imbalance of DHA, EPA, and ARA in broodstock diets reported negative effects on reproduction in several marine fish species, affecting egg and sperm quality, and decreasing fecundity, and reducing egg vitality, hatching rate and larval survival (Izquierdo et al., 2001; Furuita et al., 2003; Mazorra et al., 2003; Fernández-Palacios et al., 2011).

In conclusion, our results underline the importance of the polar lipid fraction in paralarvae lipid profile, in particular oleic acid, ARA, EPA, and DHA. The highest oleic acid content in wild paralarvae in neutral lipids also suggest the importance of this fatty acid as energy reserve, probably related to a better nutritional status in comparison with hatchlings obtained from captive broodstock. The authors would like to emphasize that the analysis of fatty acid from neutral and polar lipids provides useful information to elucidate the nutritional requirements of this species. More research must be carried out in order to understand the physiological mechanisms involved in paralarvae quality and feeding during early stages in *O. vulgaris*.

## Author contributions

JE, JS, and AM designed the experiment. BR and AL built the artificial dens and did the samplings at sea, in colaboration with JE and AM. JE did the biochemical analysis. The paper was writen by JE and AM, and revised by JS and CH, who also helped discussing the results. MI provided funding and helped discussing the results.

### Conflict of interest statement

The authors declare that the research was conducted in the absence of any commercial or financial relationships that could be construed as a potential conflict of interest.
